# Supplementary material for: Trends and characteristics of multidrug-resistant MRSA in Norway 2008-2020
Source: Front Microbiol. 2025 May 9;16:1564943. doi: 10.3389/fmicb.2025.1564943 (PMC12098411; doi:10.3389/fmicb.2025.1564943)
Supplement: Supplementary file 2 [file Data_Sheet_1.docx]

**Supplementary tables and figures**


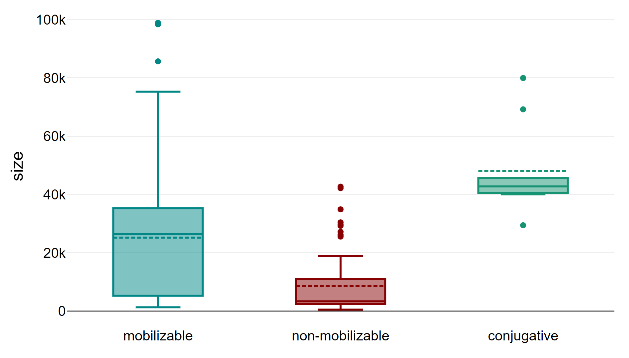


**Fig S1.** **Size of detected plasmids by predicted mobility in sequenced MDR-MRSA strains from Norway.**

**Table S2**. Plasmids identified (using MOBsuite) and percentage of genes belonging to each category (AMR, stress- or virulence-associated) per genotype (*spa*-type and CC) of sequenced MDR-MRSA strains.

|  | Plasmid | AB110 | | AB628 | | | AB631 | AB633 | AB640 | AB646 | AB722 | AB725 | AB924 | | AB973 | | AC333 | AC627 | AA003 | AA010 | AA013 | AA069 | | AA083 | | AA367 | AA379 | AA409 | | AA411 | | | AA412 | AA764 | AA770 | | AA840 | | AA841 | | AA843 | | AA848 | | | AA849 | | AA850 | | AA851 |
| --- | --- | --- | --- | --- | --- | --- | --- | --- | --- | --- | --- | --- | --- | --- | --- | --- | --- | --- | --- | --- | --- | --- | --- | --- | --- | --- | --- | --- | --- | --- | --- | --- | --- | --- | --- | --- | --- | --- | --- | --- | --- | --- | --- | --- | --- | --- | --- | --- | --- | --- |
|  | Category | AMR | STRESS | AMR | STRESS | VIR | AMR | AMR | AMR | STRESS | STRESS | STRESS | AMR | STRESS | AMR | STRESS | AMR | AMR | AMR | AMR | AMR | AMR | STRESS | AMR | STRESS | AMR | VIR | AMR | STRESS | AMR | STRESS | VIR | VIR | AMR | AMR | STRESS | AMR | STRESS | AMR | STRESS | AMR | STRESS | AMR | STRESS | VIR | AMR | STRESS | AMR | STRESS | STRESS |
| CC | *spa*-type | 20 % | 50 % | 80 % | 10 % | 10 % | 100 % | 100 % | 100 % | 100 % | 100 % | 100 % | 78 % | 22 % | 90 % | 10 % | 100 % | 100 % | 100 % | 100 % | 100 % | 56 % | 44 % | 91 % | 9 % | 100 % | 100 % | 49 % | 51 % | 48 % | 14 % | 38 % | 100 % | 100 % | 91 % | 9 % | 87 % | 13 % | 80 % | 20 % | 50 % | 50 % | 50 % | 17 % | 33 % | 30 % | 70 % | 72 % | 28 % | 100 % |
| CC1 | t127 |  |  |  |  |  |  |  |  |  |  |  |  |  |  |  | 3 | 2 |  |  |  |  |  |  |  |  |  |  |  | 61 | 7 |  |  | 6 |  |  |  |  |  |  |  |  |  |  |  |  |  |  |  |  |
|  | t14441 |  |  | 8 | 1 | 1 |  |  | 2 |  |  |  |  |  |  |  | 1 |  |  |  |  |  |  |  |  |  |  |  |  |  |  |  |  |  |  |  |  |  |  |  |  |  |  |  |  |  |  |  |  |  |
|  | t657 |  |  |  |  |  |  |  |  |  | 1 |  |  |  |  |  |  |  |  |  |  |  |  |  |  |  |  |  |  |  |  |  |  |  |  |  |  |  |  |  |  |  |  |  |  |  |  |  |  |  |
| CC188 | t189 |  |  |  |  |  |  |  |  |  |  |  |  |  | 88 | 9 | 7 |  |  | 8 |  |  |  |  |  |  |  |  |  |  |  |  |  |  |  |  |  |  |  |  | 1 | 1 |  |  |  |  |  |  |  |  |
|  | t2174 |  |  |  |  |  | 3 |  |  |  |  |  |  |  | 13 | 2 | 2 |  |  |  |  |  |  |  |  |  |  |  |  |  |  |  |  |  |  |  |  |  |  |  |  |  |  |  |  |  |  |  |  |  |
|  | t8275 |  |  |  |  |  |  |  |  |  |  |  |  |  | 6 | 1 |  |  |  |  |  |  |  |  |  |  |  |  |  |  |  |  |  |  |  |  |  |  |  |  |  |  |  |  |  |  |  |  |  |  |
| CC239 | NT | 1 | 6 |  |  |  |  |  |  |  |  |  |  |  |  |  |  |  |  |  |  |  |  |  |  |  |  |  |  |  |  |  |  |  |  |  |  |  |  |  |  |  |  |  |  |  |  |  |  |  |
|  | t030 |  |  |  |  |  |  |  |  | 1 |  |  |  |  |  |  |  |  |  |  |  |  |  |  |  |  |  |  |  |  |  |  |  |  |  |  |  |  |  |  |  |  |  |  |  |  |  |  |  |  |
|  | t037 | 1 | 2 |  |  |  |  | 2 |  |  |  |  |  |  |  |  |  | 1 |  |  |  |  |  | 2 |  |  |  |  |  |  |  |  |  |  |  |  |  |  |  |  |  |  |  |  |  |  |  |  |  |  |
|  | t275 |  |  |  |  |  |  | 1 |  |  |  |  |  |  |  |  |  |  |  |  |  |  |  |  |  |  |  |  |  |  |  |  |  |  |  |  |  |  |  |  |  |  |  |  |  |  |  |  |  |  |
|  | t632 |  |  |  |  |  |  |  |  |  |  |  |  |  |  |  |  | 1 |  |  |  |  |  |  |  |  |  |  |  |  |  |  |  |  |  |  |  |  |  |  |  |  |  |  |  |  |  |  |  |  |
| CC30 | t021 |  |  |  |  |  |  |  |  |  |  |  | 6 |  |  |  |  |  |  |  |  |  |  |  |  |  |  |  |  |  |  |  |  |  |  |  |  |  |  |  |  |  |  |  |  |  |  |  |  |  |
|  | t665 |  |  |  |  |  |  |  |  |  |  |  |  |  |  |  |  |  |  |  |  |  |  | 3 |  |  |  |  |  |  |  |  |  |  |  |  |  |  |  |  |  |  |  |  |  |  |  |  |  |  |
| CC398 | t011 |  |  |  |  |  |  |  |  |  |  |  |  |  |  |  |  | 1 | 1 |  |  |  |  |  |  |  |  |  |  |  |  |  |  |  |  |  |  |  |  |  |  |  |  |  |  |  |  |  |  |  |
| CC5 | t001 |  |  |  |  |  |  |  |  |  |  |  |  |  |  |  | 1 |  |  |  |  | 3 | 1 |  |  |  |  |  |  |  |  |  |  |  |  |  |  |  |  |  |  |  |  |  |  | 3 | 7 |  |  |  |
|  | t045 |  |  |  |  |  |  |  |  |  |  |  |  |  |  |  |  |  |  |  |  |  |  |  |  |  |  | 22 | 28 |  |  |  |  |  |  |  |  |  |  |  |  |  |  |  |  |  |  |  |  |  |
|  | t067 |  |  |  |  |  |  |  |  |  | 1 |  |  |  |  |  |  |  |  |  |  |  |  | 2 | 1 |  |  |  |  |  |  |  |  |  |  |  | 8 |  |  |  |  |  |  |  |  |  |  |  |  |  |
|  | t439 |  |  |  |  |  |  |  |  |  |  |  |  |  |  |  |  |  |  |  |  |  |  |  |  |  |  | 10 | 12 |  |  |  |  |  |  |  |  |  |  |  |  |  |  |  |  |  |  |  |  |  |
|  | t442 |  |  |  |  |  |  |  |  |  |  |  |  |  |  |  | 1 |  |  |  |  |  |  |  |  |  |  |  |  |  |  |  |  |  |  |  |  |  |  |  |  |  | 3 | 1 | 2 |  |  |  |  |  |
|  | t9408 |  |  |  |  |  |  |  |  |  | 1 |  |  |  |  |  |  |  |  |  |  |  |  | 3 |  |  |  |  |  |  |  |  |  |  |  |  | 8 | 1 |  |  |  |  |  |  |  |  |  |  |  |  |
| CC59 | t437 |  |  |  |  |  |  |  |  |  |  |  |  |  |  |  |  |  |  |  |  |  |  |  |  |  |  |  |  |  |  |  |  |  |  |  |  |  | 4 | 1 |  |  |  |  |  |  |  |  |  |  |
| CC672 | t3841 |  |  |  |  |  |  |  |  |  | 1 |  |  |  |  |  |  |  |  |  |  |  |  |  |  |  |  |  |  | 8 | 1 |  |  |  |  |  |  |  |  |  |  |  |  |  |  |  |  |  |  |  |
| CC72 | t3092 |  |  |  |  |  |  |  |  |  |  |  |  |  |  |  | 1 | 1 |  |  |  |  |  |  |  |  |  |  |  |  |  |  |  |  |  |  |  |  |  |  |  |  |  |  |  |  |  |  |  |  |
| CC8 | t008 |  |  |  |  |  |  |  |  |  |  |  | 8 | 4 |  |  |  |  |  |  |  |  |  |  |  |  | 1 |  |  |  |  |  |  |  |  |  | 30 | 6 |  |  |  |  |  |  |  |  |  |  |  |  |
|  | t064 |  |  |  |  |  |  |  |  |  |  | 1 |  |  |  |  |  | 3 |  |  | 1 | 6 | 6 |  |  | 1 |  |  |  |  |  |  |  |  |  |  |  |  |  |  |  |  |  |  |  |  |  | 22 | 8 |  |
|  | t1476 |  |  |  |  |  |  | 1 |  |  |  |  |  |  |  |  |  | 8 |  |  |  |  |  |  |  |  |  |  |  | 5 | 13 | 59 | 1 |  |  |  |  |  |  |  |  |  |  |  |  |  |  |  |  |  |
|  | t1952 |  |  |  |  |  |  |  |  |  |  |  |  |  |  |  | 1 | 1 |  |  |  |  |  |  |  |  |  |  |  |  |  |  |  |  |  |  |  |  |  |  |  |  |  |  |  |  |  | 6 | 1 |  |
|  | t451 |  |  |  |  |  |  | 1 |  |  |  | 2 |  |  |  |  | 1 | 3 |  |  |  |  |  |  |  |  |  |  |  |  |  |  |  |  |  |  |  |  |  |  |  |  |  |  |  |  |  | 3 | 3 | 1 |
| CC80 | t044 |  |  |  |  |  |  |  |  |  |  |  |  |  |  |  |  |  |  |  |  |  |  |  |  |  |  |  |  |  |  |  |  |  | 10 | 1 |  |  |  |  |  |  |  |  |  |  |  |  |  |  |
| CC9 | t337 |  |  |  |  |  |  |  |  |  |  |  |  |  |  |  |  |  |  |  |  |  |  |  |  |  |  | 8 | 1 |  |  |  |  |  |  |  |  |  |  |  |  |  |  |  |  |  |  |  |  |  |
|  | Total | 2 | 8 | 8 | 1 | 1 | 3 | 5 | 2 | 1 | 4 | 3 | 14 | 4 | 107 | 12 | 18 | 21 | 1 | 8 | 1 | 9 | 7 | 10 | 1 | 1 | 1 | 40 | 41 | 74 | 21 | 59 | 1 | 6 | 10 | 1 | 46 | 7 | 4 | 1 | 1 | 1 | 3 | 1 | 2 | 3 | 7 | 31 | 12 | 1 |
